# Recent Progress in Graphene-Based Electrocatalysts for Hydrogen Evolution Reaction

**DOI:** 10.3390/nano12111806

**Published:** 2022-05-25

**Authors:** Xupeng Qin, Oluwafunmilola Ola, Jianyong Zhao, Zanhe Yang, Santosh K. Tiwari, Nannan Wang, Yanqiu Zhu

**Affiliations:** 1 Guangxi Institute Fullerene Technology (GIFT), Key Laboratory of New Processing Technology for Nonferrous Metals and Materials, Ministry of Education, School of Resources, Environment and Materials, Guangxi University, Nanning 530004, China; qinxupeng@st.gxu.edu.cn (X.Q.); 1939200531@st.gxu.edu.cn (J.Z.); 2039200206@st.gxu.edu.cn (Z.Y.); 2 Advanced Materials Group, Faculty of Engineering, University of Nottingham, Nottingham NG7 2RD, UK; oluwafunmilola.ola1@nottingham.ac.uk; 3 Faculty of Chemistry, University of Warsaw, 1 Pasteur Str., 02-093 Warsaw, Poland; ismgraphene@gmail.com

**Keywords:** hydrogen evolution reaction, electrocatalysis, graphene, heteroatom-doped graphene, 3D structure

## Abstract

Hydrogen is regarded as a key renewable energy source to meet future energy demands. Moreover, graphene and its derivatives have many advantages, including high electronic conductivity, controllable morphology, and eco-friendliness, etc., which show great promise for electrocatalytic splitting of water to produce hydrogen. This review article highlights recent advances in the synthesis and the applications of graphene-based supported electrocatalysts in hydrogen evolution reaction (HER). Herein, powder-based and self-supporting three-dimensional (3D) electrocatalysts with doped or undoped heteroatom graphene are highlighted. Quantum dot catalysts such as carbon quantum dots, graphene quantum dots, and fullerenes are also included. Different strategies to tune and improve the structural properties and performance of HER electrocatalysts by defect engineering through synthetic approaches are discussed. The relationship between each graphene-based HER electrocatalyst is highlighted. Apart from HER electrocatalysis, the latest advances in water electrolysis by bifunctional oxygen evolution reaction (OER) and HER performed by multi-doped graphene-based electrocatalysts are also considered. This comprehensive review identifies rational strategies to direct the design and synthesis of high-performance graphene-based electrocatalysts for green and sustainable applications.

## 1. Introduction

With the expansion of the human population, the demand for energy in society is increasing and conventional fossil fuels (such as coal, oil, natural gas, etc.) cannot satisfy the needs of social development. More importantly, the use of conventional fossil fuels results in air pollution linked to the greenhouse effect [1]. Development and utilization of alternative energy sources are urgently needed; therefore, in this context, hydrogen research has received increasing attention [2,3]. Hydrogen is considered an environmentally friendly alternative to fossil fuels because its combustion product is water and its energy density is up to 140 MJ/kg [2,4,5]. Hydrogen is produced through a variety of forms (including thermal chemical technology, electrochemical technology, biotechnology, and photoelectrochemical means [6]), and the method of hydrogen fuel preparation through electrocatalytic hydrogen evolution reaction (HER) has been attracting more and more attention. This simple, efficient, and clean method for large-scale hydrogen production is now more important and promotes the rapid development of the hydrogen economy [7,8].

Electrocatalysts play a key role in these energy conversion technologies and can greatly accelerate reaction kinetics and efficiency by reducing the activation energy and thus the overpotential [9,10]. Superior HER catalysts rely on the use of platinum (Pt) [11], but large-scale industrial applications of this rare metal are very limited in terms of yield and cost. Therefore, inorganic catalysts such as metal sulfides [12,13], metal oxides [14], and metal nitrides [15] have been extensively studied as efficient, abundant, and non-expensive catalysts to replace the role of Pt in hydrogen production while promoting potential green energy sustainability on a global scale.

The core of water electrolysis reaction includes OER and HER [16,17,18], and its total chemical reactions are listed below:

In alkaline media:

At anode: 4OH^−^ → 2H_2_O + O_2_ + 4e^−^

At cathode: 2H_2_O + 2e^−^ → H_2_ + 2OH^−^

In acidic media:

At anode: 2H_2_O → 4H^+^ + O_2_ + 4e^−^

At cathode: 4H^+^ + 4e^−^ → 2H_2_

**Scheme 1:**
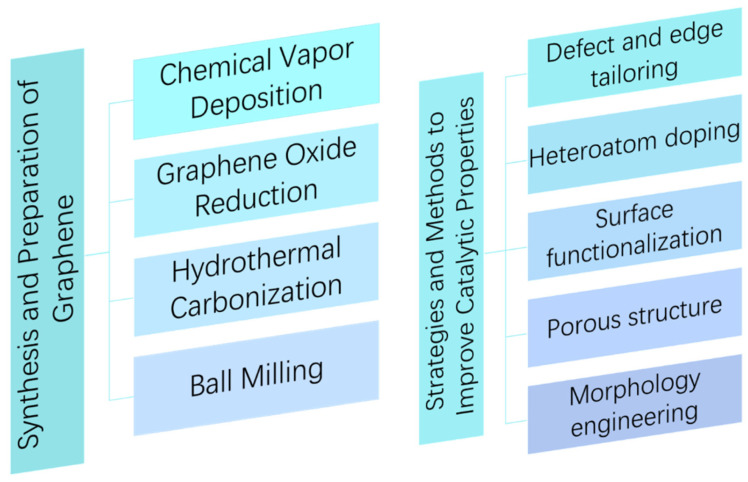
Schematic diagram of graphene electrocatalysts for HER and graphene preparation method.

The degree of electrolyte acidity has been reported to have significant impact on reaction products [19]. In the ideal case, the thermodynamic decomposition voltage of water at 25 °C and 1 atmosphere must be greater than 1.23 V before electrolysis can take place, but in actual production, the thermodynamic potential is much higher than 1.23 V. This overpotential is mainly used to overcome the inherent activation barrier on the positive and negative electrodes and overcome the kinetic barrier of reaction intermediates [17,18,20]. Comparing the overpotential required by OER and HER, the former requires a larger overpotential. The high overpotential demand of OER may be due to its complex four-electron transfer and multi-step reaction behavior, which is why the catalytic efficiency of OER is low, while HER consists of only two electron transfer processes. However, from a kinetic point of view, the hydrogen precipitation reaction still proceeds slowly, so catalysts are required for water electrolysis [21]. Common HER electrocatalysts are from the family of Pt and other precious metals [4,16], but due to their high cost and relatively small reserves, they are not practical for widespread use in a hydrogen-based economy [22]. The stability of such catalysts is also a difficult issue, so the development of new high-efficiency and stable electrocatalysts is very urgent. To overcome this, researchers have focused on modifying these materials by employing a variety of strategies. For example, heteroatom doping [23], nanoparticles encapsulation [24], defect engineering [25], surface functionalization [26], interface effects engineering [27], edge tailoring [28], 3D morphology construction [29], porous structure manufacturing [30], and other strategies can be used to further improve catalytic performance of HER electrocatalysts (Scheme 1).

Due to the exceptional physical and chemical characteristics of advanced carbon materials, the use of carbon-based materials (such as graphene [31], graphene oxide [32], carbon nanotubes [33,34], porous carbon [35], fullerenes [36], and carbon cloth [37]) as HER electrocatalysts has broad prospects. For example, Chen et al. [33] used the hydrothermal method to grow Mn-doped MoS_2_ nanosheets on carbon nanotubes (CNTs), so that the highly coupled HER catalyst had more active sites. The mass transfer process was improved and the overpotential was reduced (5% Mn-MOS_2_/CNT was 150 mV at −10 mA/cm^2^), showing excellent HER catalytic activity. In contrast, in alkaline solution, Zhang et al. [38] used a relatively simple green electroless plating method to coat the original carbon nanotubes (CNTs) with Cu_2_O nanoparticles, thus having enough active sites. At −10 mA/cm^2^, the overpotential was 101 mV and the catalyst had high long-term activity. Carbon quantum dots (CQDs) are also a new category of carbon nanomaterials that have attracted much interest from scholars in the field of electrocatalysis [39,40]. Recently, He et al. [41] devised a facile method for the electrolysis-assisted synthesis of efficient Pt and CQDs co-loaded MWCNT catalysts (Pt-CQDs/MWCNT) for the subsequent HER electrocatalysis process, and the analytical results showed that Pt and CQDs were loaded on MWCNT and the loading increased with the electrolysis voltage. CQDs acts as an abundant nucleation and anchoring site in this electrocatalyst, improving the immobilization and dispersion of Pt NPs. CQDs can build a 3D conductive network with MWCNT through π-π bonding, thus exhibiting a smaller charge transfer resistance and faster reaction rate, which is conducive to improved electrocatalytic performance and long-term stability. Similarly, Sayed et al. [42] also exploited the excellent stability and unique structure of nitrogen-doped metal-free catalysts by incorporating AgNi alloy nanoparticles on nitrogen-doped CQDs to produce a new electrocatalyst that provides more active sites for electrocatalytic water decomposition as the temperature increases, resulting in a significant increase in the catalytic activity of the sample. Catalytic activity of the samples also increased significantly. Alain R’s team [36] constructed van der Waals 1T-MoS_2_/C60 zero- to two-dimensional (0D–2D) heterostructures by a one-pot synthesis for HER. The interfacial interactions of 1T-MoS_2_-C60 and C60-C60 and their electrochemical catalytic properties can be well-managed by varying the weight percentage of fullerenes, and the heterostructure domain NSs of 1T-MoS_2_ and C60 were shown to have good HER properties.

As carbon-based electrocatalysts continue to develop, the future will be geared towards higher yields, lower prices, and a more environmentally friendly approach. Among these different materials, graphene-based materials, which include graphene and its derivatives, i.e., reduced graphene, GQDs, functionalized graphene, and doped graphene as the electroactive components, show great promise for water electrolysis applications and are of great interest to scientists in the field of catalysis, considering their intrinsic properties.

With the rapid development of the hydrogen energy industry, graphene and its derivatives are notable for their unique properties and they have become an unmissable representative in the field of HER, playing an integral role in the rapid development of electrocatalysis. Graphene, a 2D layered nanomaterial with a hexagonal dotted structure formed by the hybridization of carbon atoms in sp^2^ orbitals, was produced by Andre Geim and Konstantin Novoselov in 2004 by mechanical exfoliation [43], for which they were jointly awarded the Nobel Prize in Physics. Each carbon atom in the two-dimensional structure of graphene is tightly bound to neighboring carbon atoms through a unique electron cloud [44]. Graphene currently belongs to a unique class of zero-bandgap nanomaterials, often regarded as semi-metallic materials [45], in which each carbon atom is sp^2^ hybridized. Single-layer graphene is reported to be able to withstand stresses of up to 42 N/m and has a Young’s modulus of 1.0 TPa [44,46], properties that make graphene materials of great potential for aerospace applications. In addition, one of the most important properties of graphene is its very high electrical conductivity, where each atom in the two-dimensional structure is attached to three other carbon atoms in the two-dimensional plane by interaction, while the remaining one electron acts as a component of the π-bond. Its intrinsic carrier mobility can reach 200,000 cm^2^·V^−1^·s^−1^ under some critical conditions [47], the electrons and holes of graphene are known as Dirac fermions [44,46], and based on this property, scientists have used it extensively in the preparation of catalysts for the electrochemical decomposition of water.

Considering the recent advances in the role of graphene in water electrolysis, this review aims to highlight the latest developments in non-precious-metal-based graphene catalysts for HER. In later chapters, we focus on the latest applications of non-precious-metal-based graphene electrocatalysts in HER, including flake graphene, heteroatom-doped graphene, graphene aerogels, 3D graphene skeletons, quantum dots, carbon nanotubes, fullerenes, etc. We conclude with our views and understanding of the current progress, future trends, and challenges of non-precious-metal-based graphene catalysts.

## 2. Synthesis and Preparation of Graphene

### 2.1. Chemical Vapor Deposition (CVD)

Low-dimensional carbon nanomaterials in a variety of forms play an essential role in new synthetic materials, and therefore the development of new low-dimensional carbon materials has received a great deal of attention from researchers. There are many methods to prepare new low-dimensional carbon materials, among which CVD is a commonly used preparation method due to its many advantages of simple operation, controlled product morphology, and high purity. CVD has been widely used to prepare graphene, carbon nanotubes, graphene nanoribbons, etc. [48]. The chemical vapor deposition procedure consists of three significant stages: the entry of the gas to be reacted onto the substrate surface, the adsorption of the reacting gas onto the substrate surface, the deposition of the product onto the substrate surface, and the release of the resulting vapor phase byproducts. CVD technology has become an important approach for the large-scale fabrication of large-sized and high-quality graphene for a number of purposes.

CVD techniques can be divided into two categories [49]: the first with metal-catalyzed growth, which suffers from disadvantages including relatively high metal consumption, limited metal microstructure options, and unavoidable metal residues in the product; and the second is direct metal-free catalyst growth. For example, Wei et al. [50] report a metal-free CVD process under CH_4_ + NH_3_ plasma conditions that produces monolayer nitrogen-doped graphene (NG) immediately on amorphous insulating SiO_2_/Si surfaces as well as on Al_2_O_3_, h-BN, and mica. In addition, Li et al. [51] used methane CVD to generate high-quality graphene on oxide surfaces in the absence of a metal catalyst.

**Figure 1:**
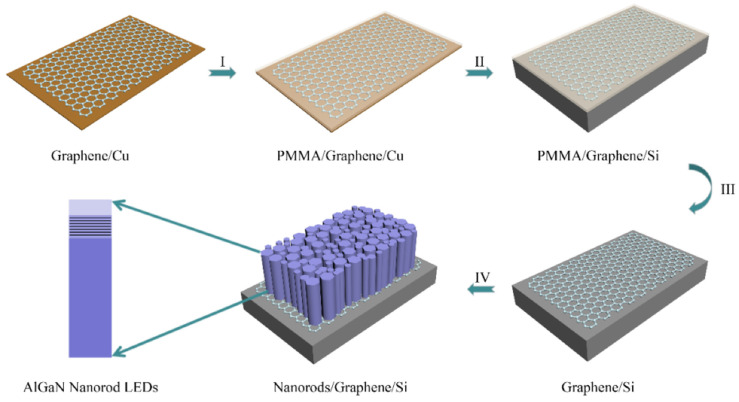
Schematic representation of the graphene transfer procedure and the key processes involved in metal organic chemical vapor deposition (MOCVD) for growing AlGaN nanorod LEDs. (I) spin coating of poly methyl methacrylate (PMMA) on graphene on Cu foil; (II) the transfer of graphene with PMMA to Si substrate; (III) dissolution of PMMA; and (IV) MOCVD growing of AlGaN nanorod LEDs. Reprinted with permission from Ref. [52]. Copyright 1996–2022 MDPI (Basel, Switzerland).

As shown in Figure 1, metal organic chemical vapor deposition (MOCVD) under metal-free catalyst conditions was performed by Ren et al. [52]. This resulted in the growth of AlGaN light-emitting diodes (LEDs) directly on Si substrates covered with a single layer of graphene.

### 2.2. Graphene Oxide Reduction

Graphene oxide is an oxide of graphene and a new type of carbon material, generally denoted by GO, and is commonly available in powder, flake, and solution form. It is a monolayer or several-layer structure obtained by oxidation and ultrasonic treatment of graphite, which can be readily extended in lateral dimensions to tens of microns and is one of the more representative graphene derivatives. The structure of graphene oxide (GO) spans the typical scales of general chemistry and materials science, with properties of polymers, colloids, thin films, and amphiphilic molecules. Research into GO has therefore never stopped since it was found in 2004 by Andre Geim and Konstantin Novoselov.

In the preparation of graphene oxide, Hummer used potassium permanganate and concentrated sulfuric acid to oxidize graphite to synthesize graphite oxide, historically known as Hummers’ method [53]. Since then, a large number of scientists have continued to improve on the Hummers method, for example, Chen et al. [54] have eliminated the use of NaNO_3_ in the process, thereby eliminating the production of toxic gases, simplifying the purification of the waste stream and further reducing costs to facilitate mass production.

**Figure 2:**
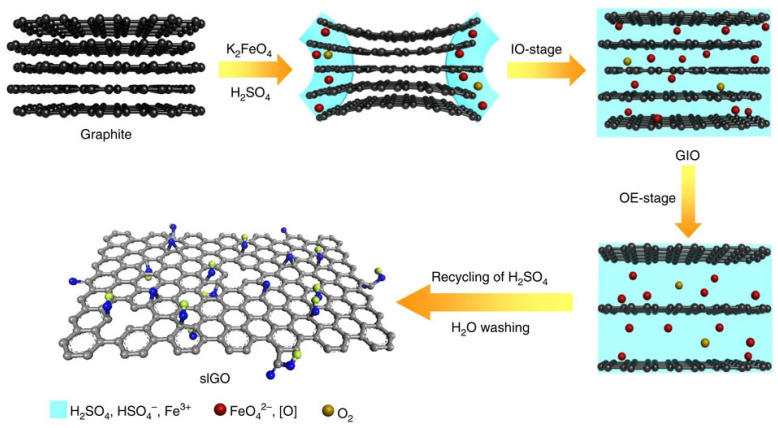
There are two key steps in this preparation process: intercalation-oxidation (IO) and oxidation-exfoliation (OE). In the IO step, concentrated sulfuric acid and oxidant are intercalated into the graphite layer to form intercalated graphite oxide (GIO). During the intercalation process, the oxidizer breaks the π-π conjugate structure of graphite, creating negatively charged functional groups and increasing the interlayer spacing. In the next OE stage, the oxidizing agent further oxidizes the carbon basal plane of GIO, producing more functional groups and expanding the interlayer space. After recovery of sulfuric acid and cleaning with water, 100% slGO was attained (Blue and green balls are O and H, respectively). Reprinted with permission from Ref. [55]. Copyright 2022 Springer Nature Limited.

The green and non-polluting method of producing high-quality monolayer GO in 1 h was proposed by Peng et al. [55], as shown in Figure 2. On the one hand, heavy metals and toxic gas interference are reduced, and on the other hand, sulfuric acid can be recovered.

It is worth noting that GO can also be used to manufacture graphene on a large scale, and that the GO reduction method consists of two main steps. Firstly, the graphite oxide is exfoliated between the layers to obtain GO, and secondly, a portion of the oxygen-containing functional groups is removed and reduced to obtain the target product. Methods for the preparation of reduced GO can be divided into three categories: chemical reduction, thermal reduction, and electrochemical reduction. Pankaj Tambe [56] came up with a new idea of using sodium hydroxide, which is a base, to treat the acidified GO to make graphene. In the experiment, two methods were used to make GO. In this process, acid was added to acidify GO, and it was found that the spacing of acidified GO decreased. This indicates that the surface functional groups of RGO have been removed. In general, GO reduction is considered to be the most promising method of graphene preparation due to its low cost and simplicity of operation [44], but there are also other methods of preparation such as steam treatment, microwave reduction, laser, and other methods to produce reduced graphene oxide.

### 2.3. Hydrothermal Carbonization

The hydrothermal carbonization (HTC) method uses organic matter or carbohydrates as the raw material and water as the reaction medium to produce the corresponding carbon material in a hydrothermal reactor at a certain temperature and pressure, simulating the formation of coal through a series of complex reaction processes [57,58]. Depending on the working temperature [59], HTCs can be divided into two major groups: (1) high-temperature HTCs, which can synthesize carbon with a high level of graphitization, for example, carbon nanotubes or activated carbon; (2) low-temperature HTCs. Compared with other methods of carbon material preparation, the carbon material produced by HTC has the characteristics of adjustable structure and small and uniform particle size [60]. For example, Yang et al. [61] synthesized a range of size-tunable hard carbon microspheres by HTC of glucose in the presence of a little salt. The prepared carbon microspheres exhibit low cost, high capacity at low potential, outstanding multiplicative properties, and excellent cycling stability. In addition, Hu et al. [62] prepared self-supported Ni_3_Se_2_@NiFe-LDH/NF catalysts by hydrothermal heating at 160 °C for 24 h and electrodeposition. The analysis showed that the catalysts had good electrical conductivity, large surface area, good electron transfer capacity, and abundant active sites, and showed good HER electrocatalytic activity in alkaline media. Qian et al. [24] synthesized ultrathin N-doped graphene-coated Ni nanoparticles and self-supported MoO_2_ nanosheets on 3D nickel foams by hydrothermal and high-temperature post-treatment, which exhibited low overpotential and maintained long-term chemical stability in electrochemical decomposition of aqueous HER.

**Figure 3:**
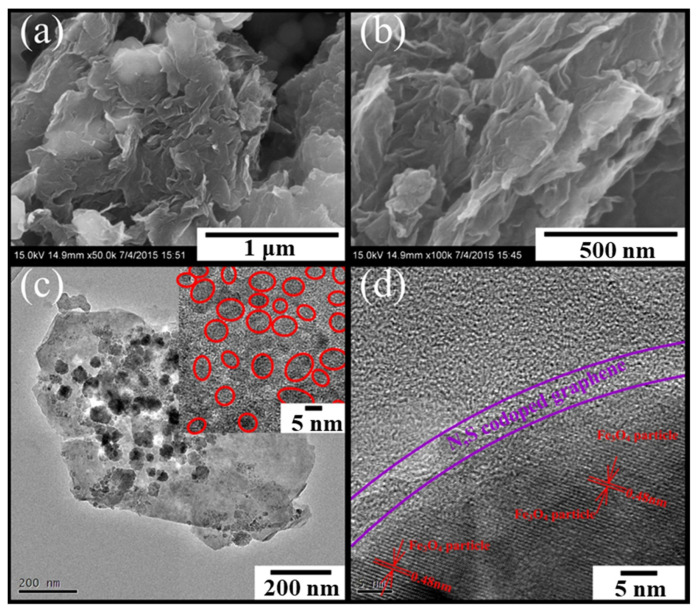
FE-SEM and TEM images of the synthesized NSG/Fe_3_O_4_ composites: (**a**) FE-SEM images reveal Fe_3_O_4_ nanoparticles encapsulated in N, S co-doped graphene nanosheets; (**b**) FE-SEM images; (**c**) TEM images show that there are many Fe_3_O_4_ nanoparticles distributed and encapsulated in N, S co-doped graphene nanosheets; (**d**) high-magnification TEM images showing that Fe_3_O_4_ nanoparticles are contained in N, S co-doped graphene nanosheets effectively. Reprinted with permission from Ref. [65]. Copyright 2022 Springer Nature Limited.

In another study of three-phase hybridization, Xiao et al. [63] prepared 1T/2H-MoS_2_/RGO heterostructured hybrids using a one-step hydrothermal method maintained at 200 °C for 30 h. On the one hand, due to the transverse heterogeneous structure of the 1T/2H-MoS_2_ sheet, and on the other hand, due to the coupling function between the RGO support sheets, the RGO can effectively suppress the 1T-2H phase transition, thus further improving the stability of this catalyst. The influence of hydrothermal conditions at different temperatures on the preparation of materials was investigated. The optimal preparation temperature was 200 °C, which indicated that the catalyst had higher stability. The specific N,S co-doped graphene/Fe_3_O_4_ hybrid materials were finally synthesized by a combination of hydrothermal process and carbonization heat treatment by Yang et al. [64], as illustrated in Figure 3.

### 2.4. Ball Milling

Ball milling is a top-down, green, mechanochemical method that uses the impact of falling grinding bodies and the grinding action of the grinding bodies against the inner wall of the ball mill to crush and mix materials [65]. In this process, a specific powder is placed with the grinding medium and the kinetic energy generated during the movement of the moving ball is applied to the powder charge, thereby breaking the chemical bonds of the molecules involved and reducing the particle size. Some progression occurs through the transfer of mass and energy as well as mechanical stresses due to milling, leading to the destruction of the lattice structure of the material [66]. Ball mills are inexpensive to install and can grind media for continuous operation, but avoiding contamination from the grinding media is an important issue. At the same time, many parameters control various aspects of ball milling, for example the type of ball mill (e.g., planetary ball mill, rotary ball mill, vibratory tube mill, and grinder) determines the kinetic energy intensity of the balls transferred to the powder. In addition, there is the mill speed and temperature, as well as the ball size and load.

Today, ball mills are widely used in the synthesis of nanomaterials. Francy’s team [67] prepared graphene oxide (GO) using a ball milling method with potassium perchlorate as the oxidant and deionized water as the grinding medium, and compared it with samples produced by the Hummers method. The results of the analysis showed the feasibility of the method. Xu’s team [68] successfully synthesized N-doped biochar by simply ball milling virgin biochar with ammonium hydroxide. N such as amine (-NH_2_) and nitrile (C≡N) produced by the dehydration of O-containing functional groups such as COOH and OH is loaded on the surface of the biochar. A new method for the synthesis of N-doped carbon materials is provided. The low-cost manufacturing of GNs is of great importance for practical purposes. In-Yup Jeon’s team [69] enabled high yields of edge-selective carboxylated graphite without chemical hazards by simply ball milling pristine graphite under dry ice conditions, and confirmed the reaction mechanism through various microscopic and spectroscopic measurements.

## 3. Strategies and Methods to Improve Catalytic Properties

### 3.1. Heteroatom Doping

The use of heteroatomic doping on graphene electrocatalysts can create more active sites and promotes the electrochemical properties of the active sites. Numerous studies have shown that enriching the heteroatom content of graphene can significantly improve its HER activity. Due to the different electronegativity of heteroatoms and graphene, different heteroatoms change the electronic structure of sp^2^-hybridized graphene, causing charge polarization in graphene and leading to rearrangement of the electronic structure of graphene, which in turn can lower the potential barrier of the chemical reaction and change the reaction kinetic process to show better catalytic reaction properties. For example, Zhang et al. [70] used urea together with graphene oxide for thermal annealing treatment, resulting in the spontaneous transformation of graphene oxide into nitrogen-doped graphene, while further dissolving iridium (Ir) and cobalt (Co) to prepare NG@IrCo/NG composite catalysts, which showed good catalytic activity during the electrochemical decomposition of water.

Sun et al. [71] prepared Ca1-NG non-homogeneous catalysts using graphene oxide (GO) and CaCl_2_ in NH_3_ atmosphere, in which N, Ca, and C elements were homogeneously doped in the graphene lattice, with a minor Tafel slope and an overpotential of 151 mV at a current density of 10 mA/cm^2^.

**Figure 4:**
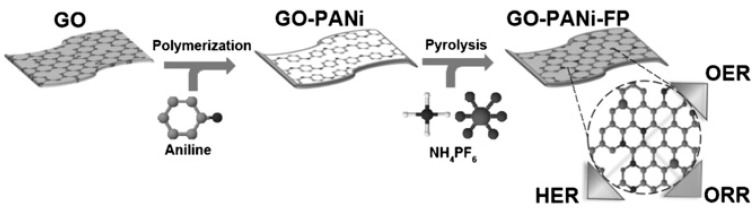
Preparation method of GO-PANi-FP tri-functional electrocatalyst material. Reprinted with permission from Ref. [72]. Copyright 2016 John Wiley and Sons.

Zhang et al. [72] prepared a versatile electrocatalyst consisting of nitrogen, phosphorus, and fluorine tri-doped graphene, where N, P, and F were obtained by pyrolysis of poly-aniline (PANi)-coated GO (GO-PANi) with NH4PF6, as shown in Figure 4.

Later in this article, the latest advances in single-atom doped graphene for the promotion of HER, including N, S, P, B, and many other non-metallic dopants, as well as the latest advances in double-atom doped graphene-based HER electrocatalysts, will be described in detail.

### 3.2. Surface Functionalization

An important challenge when using carbon-based materials as electrocatalysts is the hydrophobicity of the carbon-based material. To increase the contact area for better dispersion in the electrolyte, surface functionalization is particularly important. Heteroatoms such as N, B, O, and S are doped by introducing substances with oxygen functional groups such as hydroxyl, epoxy, and carboxyl groups, or by oxidizing the graphite lattice to form defective sites [73]. For example, oxygen-containing groups, such as ketones, phenols, lactones, carboxyl groups, ethers, anhydrides, etc., are usually introduced to the surface of carbon nanotubes by controlled oxidation. The introduction of oxygen-containing groups can ameliorate the hydrophilicity or wettability of the CNT surface in polar solvents such as water, and on the other hand these oxygen-containing groups can serve as anchor points for subsequent functionalization [74]. In the case of graphene oxide, the periphery is mainly modified by carboxylic acids, anhydrides, acetone, and phenolic groups, while the base of the GO sheet is controlled by epoxy groups and hydroxyl groups. These oxygen-containing groups are essential for catalytic activity when modifying oxygen-containing functional groups and carbon vacancies; the configuration of the basal plane and the planarity GO of pure sp^2^ will be lost. This allows for an increase in active sites, which can significantly enhance catalytic activity, and the activity of these chemically modified graphene-based materials depends on the type and number of functional groups introduced [75]. The unique structural properties of graphene allow the application of modification and functionalization strategies to improve performance, thus broadening its application in catalysis.

### 3.3. Defect and Edge Tailoring

Two-dimensional materials inevitably have disorderly or intrinsic defects, the simplest and most abundant of which are vacancies, and there are generally two types of defects, namely point defects as well as line defects, the presence of which breaks structural symmetry and alters the partial distribution of electrons, thus facilitating the enhancement of electrochemical activity [76,77]. Defects play an essential role in the electrochemical decomposition of the HER, oxygen reduction reaction (ORR), and OER of water. From a kinetic point of view, defect sites can be used as active sites for hydrogen evolution, and the introduction of defect structures can promote the HER process by optimizing the electronic structure of the catalyst.

Heteroatom doping is a practical method to increase the defect density, which is very effective in tuning the energy band structure and electrical properties of 2D materials. Tian et al. [78] proposed an effective strategy to exploit the potentially high HER activity of graphene by defect engineering with heteroatoms. P-NSG electrocatalysts were prepared using microwave-assisted thermal methods and plasma etching. The analytical results show that the synergistic combined effect of N and S co-doping and plasma-induced structural defects can maximize the exposed activated sites and dramatically increase the catalytic activity of graphene in the electrochemical decomposition of aqueous HER. In order to significantly improve the defect density of nickel-based catalysts using defect engineering, Zhang et al. [79] synthesized a new microtubular electrocatalyst consisting of carbon flakes and Ni nanoparticles and controlled the defect density by adjusting the heating method and time. With these remarkable features, the prepared electrocatalysts showed significantly improved HER electrocatalytic activity and stability.

In addition, edge modulation plays a crucial role in the catalytic activity of graphene materials [80]. Compared to the basal plane, the edge sites are significantly more reactive than the basal plane and have a significant influence on the electronic characteristics and surface reactivity [81]. Graphene’s edges are reported to be twice as reactive as most atoms and have a faster electron transfer rate [82].

Based on the edge position theory, Akichika et al. [83] constructed N-doped graphene, P-doped graphene, and NP co-doped graphene with edge-enriched architecture by CVD. Raman spectroscopy analysis showed that the density of atomic-level defects is highly concentrated near the edges and lower in regions away from the edges; the architecture accumulates chemical dopants near the graphene edges and adjusts the HER activity configuration. The defect-free graphene layer materials in relatively low-dose planar regions were found to enhance the reaction kinetics by supporting electron transport to the edge regions, enhancing the reaction kinetics and facilitating the HER process for the electrochemical decomposition of water.

### 3.4. Porous Structure and Morphology Engineering

In simple terms, the porous structured nanomaterials are useful for catalysis because of their large surface area and the fact that they can play an essential role in enhancing electrolyte penetration and promoting ion diffusion, significantly shortening the electron transport path. The 3D porous nanomaterial structure offers a large Brunauer–Emmett–Teller surface area with a number of exposure reaction sites. The 3D conductive carbon networks and cross-linked structures prepared from graphene-based materials provide ample electron transport capabilities and the construction of 3D graphene networks greatly improves mechanical stability.

Three-dimensional graphene is considered to be the best scaffold to enhance the electrocatalytic HER activity of supported nanomaterials. To this end, three-dimensional nitrogen-doped graphene-coated Ni_2_P/Ni_5_P_4_ heterogeneous nanoparticle hybrid catalyst (Ni_2_P/Ni_5_P_4_@3DNG) was prepared by Ding et al. [84]. The analytical results showed that due to the 3D porous structure providing more carriers for N-doping, the catalyst exhibited a low overpotential and Tafel slope in the electrolyte of 0.5 M H_2_SO_4_.

Recently, the discovery of metal organic frameworks (MOFs) has attracted much interest in HER due to their ability to be prepared as a self-templating method with a surface area much higher than the majority of carbon materials and the ability to improve conductivity by the addition of conductive materials such as transition metals. Yan et al. [85] reportedly synthesized a freestanding 3D heterostructured film (CGHF) on a nickel-centered metal organic framework (MOF)/graphene oxide. The precisely controlled composition of the MOF enabled the successful preparation of a multifunctional heterostructured film with more defective active sites and good transfer pathways to the electrolyte and ions, which resulted in enhanced electronic conductivity and fast mass charge transfer efficiencies. Significant catalytic activity for both HER and OER was observed, with low overpotentials of 95 and 260 mV at a current density of 10 mA/cm^2^, respectively.

## 4. Graphene-Based HER Electrocatalysts

### 4.1. Non-Doped Graphene-Based

#### 4.1.1. Powder Catalysts

A summary of the catalytic performance of typical graphene-based electrocatalysts in the article is presented in Table 1.

Laminated graphene, the most common shape of graphene, has received a significant amount of attention from investigators in the field of electrocatalytic decomposition of water due to its special structural properties [44]. However, as the catalytic performance of pristine graphene is almost “zero”, researchers often combine laminated graphene with various HER active materials to increase the number of active sites and thus increase water catalytic activity.

For example, Yan et al. [86] designed a novel HER electrocatalyst by vertically anchoring ReSe_2_ nanosheets on rGO using the hydrothermal method of synthesis. The ReSe_2_@rGO electrocatalyst which has active sites exposed in the vertical direction of ReSe_2_ nanosheets exhibited an overpotential of 145.3 mV and Tafel slope of 40.7 mV/dec at a current density of 10 mA/cm^2^. Hu et al. [87] investigated the relationship between the overpotential required for graphene-covered HER electrocatalysts and the proton penetration current in order to understand what factors influence graphene-covered HER catalysts. The results show that the extent of proton penetration decreases as the number of layers of graphene-covered HER catalysts increases, and that the capability of protons to penetrate graphene determines the HER activity of graphene-covered non-precious metal catalysts.

In addition, Iwona et al. [88] deposited Ni on reduced graphene oxide, which has a cauliflower-like structure with a well-developed surface morphology, thereby enhancing the catalytic activity of Ni for HER by water. A new catalyst of Ni/NiO nanoparticles encapsulated in graphene layers was prepared by Wang et al. [89] using an arc discharge method at 30 V and 100 A. The catalyst showed superior HER activity based on synergy and achieved a low overpotential of 112 mV at a current density of 20 mA/cm^2^ in 1 M KOH solution, and the HER activity equivalent to that of the rare metal Pt.

Dhana Sekaran et al. [90] prepared 2H-1T MoS_2_ nanosheets using a simple sol-gel method and further modified them on RGO to obtain a new HER electrocatalyst with a high surface area and thus more active sites; RGO has a Δ_GH_ of −0.01 eV which also implies better catalytic activity. Zhu et al. [91] prepared a new inorganic catalyst Ni_0.85_Se/RGO by a facile one-step hydrothermal method and investigated the mechanism of its increased catalytic activity. In addition, RGO facilitates the dispersion of the Ni_0.85_Se catalyst nanospheres, increasing their surface area to allow for more active sites, resulting in significant catalytic activity of 128 mV overpotential at a current density of 10 mA cm^−2^ and long-term stability of 18 h.

#### 4.1.2. 3D-Type Catalysts

In addition to the fundamental characteristics of 2D graphene, for example, its large theoretical special surface area and excellent carrier mobility, the 3D graphene network has an interconnected, hierarchical structure, extremely low mass density, and excellent mechanical stability [92,93,94,95].

Molybdenum disulfide (MoS_2_) has a unique layered structure characteristic and electron distribution with a Gibbs free energy close to zero, making it a potential HER electrocatalyst. However, the rapid development of MoS_2_ is hampered by its poor electrical conductivity and the small number of active sites present. Based on this, Saikat et al. [96] synthesized rGO-CNT/MoS_2_ with a 3D support structure that exhibited good catalytic performance in acidic or basic environments. Overpotentials of 88 mV and 172 mV were observed in acidic and alkaline media at a current density of 10 mA/cm^2^, respectively. Analysis of the structures showed that the synergistic effect of these two different carbonaceous conducting scaffolds improved the HER catalytic efficiency by reducing the charge transfer resistance. In addition, Sunil et al. [97] used a hydrothermal method to homogeneously anchor MoS_2_-WS_2_ nanoparticles on graphene to synthesize HER electrocatalyst with a 3D porous structure, characterized by a large specific surface area of graphene and reduced aggregation in TMDs nanosheets, while exposing a high number of active sites.

**Figure 5:**
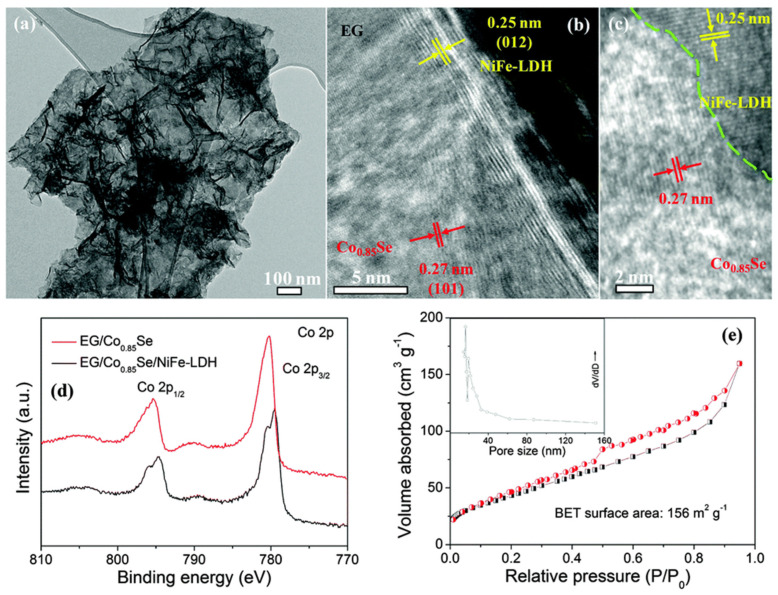
TEM (**a**) and HRTEM (**b**,**c**) images of EG/Co_0.85_Se/NiFe−LDH. (**d**) High-resolution Co 2p XPS spectra of EG/Co_0.85_Se and EG/Co_0.85_Se/NiFe−LDH. (**e**) N_2_ adsorption isotherm and the corresponding pore size distribution (inset) of EG/Co_0.85_Se/NiFe−LDH. Reprinted with permission from Ref. [98]. Copyright 2016 The Royal Society of Chemistry.

Hou et al. [98] constructed ternary hybrids by in situ growth of vertically oriented cobalt selenide (Co_0.85_Se) nanosheets on electrochemically exfoliated graphene foils followed by hydrothermal deposition of NiFe layered double hydroxides (Figure 5). The resulting 3D layered heterostructures with high surface area and strong coupling effect exhibit excellent catalytic activity toward OER and also effectively catalyze HER in bases.

Van-Toan et al. [99] developed a graphene-encapsulated 3D Pd nanosponge (Pd@G-NS) using the role of Br-ions in accelerating the stripping process of carbon dots (Cdot). The prepared Pd@G-NS had significant HER catalytic activity in acidic media and long-term stability of over 3000 cycles. Pd@G-NS showed an overpotential of 32 mV at 10 mA·cm^−2^ current density, and a Tafel slope of 33 mV·dec^−1^. In addition, Yu et al. [100] also developed a new 3D sponge structure by first reacting Ni^2+^ with a ligand to form a rectangular rod-shaped Ni-MOF, which was then calcined and graphitized. The escape of nickel nanocages to form graphene nanocages and the interaction of N and C atoms led to the formation of Ni SAs, consisting of a porous sponge-like structure assembled from Ni@C and Ni SAs in an SGNC matrix. The analysis shows that the Ni center reduced the potential barrier during hydrolysis and that the Ni NPs provide more active sites such that the composite catalyst exhibits efficient catalysis in alkaline environments, exhibiting an overpotential as low as 27 mV at a current density of 10 mA·cm^−2^.

Pt-based composites are traditionally highly efficient HER electrocatalysts with excellent catalytic properties, but unfortunately, they are expensive and have poor stability in acidic environments. Due to the similar bonding energy of Ru-H and Pt-H bonds, ruthenium (Ru) is regarded as a possible alternative to platinum-based HER electrocatalysts. To this end, Li et al. [101] designed a 3D hollow spherical structure to form SiO_2_@GO nanospheres by encapsulating the GO nanosheets with SiO_2_ nanospheres through electrostatic interactions, which led to the fabrication of Ru_x_P@NPC/GHSs hybrids. The heterogeneous carbon layers and hollow spherical structures were shown to prevent the aggregation of nanoparticles and to promote charge transfer and exposure of more active sites, thus exhibiting good catalytic performance. Ru_x_P@NPC/GHS exhibited an overpotential as low as 25.5, 60.6, and 113 mV under alkaline, acidic, and neutral environments, correspondingly, at a current density of 10 mA cm^−2^.

In addition, graphene aerogels are open-structured porous materials consisting of graphene sheets linked together by chemical bonding and van der Waals interactions. To date, many approaches have been applied to the fabrication of graphene aerogels, such as chemical vapor deposition, electrochemical reduction, template-guided preparation, and in situ self-assembly. Graphene aerogels are not only characterized by the hydrophobicity, large specific surface area, and exceptional chemical and thermal stability of graphene itself [102,103,104,105,106], but also by the large specific surface area of the aerogel’s 3D porous structure, which provides more exposed active sites. Many researchers have focused on this special 3D graphene-based structure in the hope of developing more catalytically active HER electrocatalysts.

Based on this, Li et al. [107] used a solvothermal method to electrochemically deposit a small amount of Pt nanoparticles on 3D tungsten oxide/reduced graphene oxide aerogels (WGA) to prepare a low-content Pt aerogel HER electrocatalyst with significant catalytic activity for HER catalysis. The electrocatalyst showed excellent long-term stability, exhibiting a low overpotential of 42 mV at a current density of 10 mA·cm^−2^. This is due to the interconnected porous structure materials in the 3D graphene aerogel facilitating the transfer of electrons, and the abundance of surface area in the 3D structure allowing for more active sites for Pt relevant for HER.

Cao et al. [108] synthesized poly allylamine (PA) functionalized ultrafine Rh nanoparticles (Rh-uNPs) by surface adsorption-chemical reduction and further anchored (Rh-uNPs) on graphene aerogel (GA) to obtain (PA@Rh-uNPs/GA) nanocomposites, in which the introduction of GA greatly improved the utilization of Rh to enhance the catalytic efficiency of electrolytic water. The introduction of GA greatly improved the catalytic efficiency of HER, and the PA@Rh-uNPs/GA-5000 nanocomposites containing 10% Rh mass had stronger catalytic performance and long-term stability compared to the conventional commercial 20% Pt/C electrocatalysts.

Similarly, Wei’s group [109] synthesized graphene aerogels (NiCoP-NPs@GA) embedded with NiCoP nanoparticles via the phosphorylation route using algal biomass as the reaction precursor. The electrocatalyst exhibited significant catalytic performance with an onset potential as low as 34 mV and an overpotential as low as 109 mV at a current density of 10 mA/cm^2^ compared to the NiCoP bulk.

Hyeonggeun et al. [110] hierarchically arranged 2D MoS_2_ nanosheets on 3D rGO aerogels by one-pot hydrothermal synthesis, which exhibited better catalytic performance in the hydrolysis of HER due to the porous network and more active sites in 3D reduced graphene oxide aerogels. Similarly, Rajendra et al. [111] also introduced CoS_2_ in graphene aerogels by hydrothermal method to obtain a composite CoS_2_/graphene aerogel electrocatalyst with an overpotential as low as 160 mV at a current density of 10 mA·cm^−2^.

### 4.2. Doped Graphene-Based Electrocatalysts

Intrinsic graphene exhibits strong inertness and is essentially unable to be used directly as a catalyst for HER. However, heteroatom-doped electrocatalysts based on intrinsic graphene groups can tune the electronic architecture and chemical activity of intrinsic graphene to the extent that more active sites are generated on the graphene electrocatalyst or provide anchor sites for other active substances, which can effectively improve its catalytic inertness [31,112].

#### 4.2.1. Single-Element Doped Graphene Powder Catalysts

Since N-doped graphene carriers can be used to prevent agglomeration or detachment of nanoparticles in the pyrolysis process and are beneficial in preserving the integrity of the composite [112], nitrogen atom doping is considered to be the most promising of the single atom doping types to improve activity.

Li et al. [113] produced uniformly distributed Ru nanoclusters (Ru-NG) in N-doped graphene by a two-step thermal decomposition using glucose as the carbon source, dicyandiamide as the carbon source, and RuCl_3_ as the Ru source. Ru-based nanoclusters were surrounded by N atoms and Ru-NG exhibited a low overpotential of 25.9 mV when the current intensity was 10 mA·cm^−2^ in alkaline medium. In addition, Yang et al. [114] proposed a new strategy, the “balancing effect”, whereby the interaction of TMC (M = Mo, W, Ti, and V) with H* can be weakened by nitrogen-doped graphene (NG), which balances the electronic structure, leading to the preparation of TMC@NG electrocatalysts with low Tafel slope and low overpotential. Using a solvothermal method, TMC@NG electrocatalysts possessed a bigger specific surface area to expose more active sites and lower the response barrier for the H* adsorption and desorption steps, facilitating hydrogen production.

Wang et al. [115] used pyrolysis to confine Co nanoparticles confined by N-doped rich graphene. Pyrolysis was observed to increase the interaction caused by the abundance of defects and the transfer of electrons in graphene that gives it high catalytic activity. They employed this active catalytic material in a rechargeable zinc-air battery yielding a maximum power intensity of up to 205 mW/cm^2^ and a 667 h cycle lifetime. This route to building defective N-doped carbon shell layers offers a new approach to enhancing HER.

In another study, Deng et al. [116] loaded a new Ni_2_P-microporous nickel phosphite on the surface of nitrogen-doped graphene by hydrothermal and phosphorylation methods, where the two-dimensional NGO provided more active sites and stronger loading capacity for the new electrocatalyst.

On the other hand, Liu et al. [117] investigated the effect of different P mono-doped graphene (PDGLs5) on the catalytic activity of HER and OER and showed that PDGLs5 obtained under argon atmosphere protection at 1300 °C exhibited an overpotential of −0.303 V at 10 mA·cm^−2^ and showed higher HER catalytic activity than most heteroatomic dopants. Moreover, Liu et al. also confirmed by DFT calculations that the defects arising from the decomposition of the C_3_-P=O group are the main factor in the generation of HER active sites with enhanced catalytic activity.

In addition to the typical doping components such as N and P, elements such as B and S also have special properties in doping graphene-based electrocatalysts. For example, Zhang et al. [118] studied the properties of a new two-dimensional bilayer 2L-SnP_3_/BG catalyst composed between B-doped graphene and two-dimensional layered SnP_3_, and showed that of the six doping sites, the second site was the site with the lowest structural stability and energy among the B-atom-doped 2L-SnP_3_/G nanostructures. Also in this study, Zhang et al. found that B heteroatom doping exhibited higher catalytic activity than N-atom-doped HER catalysts in a series of nL-SnP_3_/BG intercalation structures.

Jiang et al. [119] synthesized a novel self-supporting HER catalyst, FeCoNiB@B-VG, by chemically coating FeCoNiB alloy nanoparticles on B-doped perpendicularly aligned graphene alignments by chemical plating. The synergy between the materials was maximized such that more active sites were added and the conductivity was increased. Using 1.0 M KOH and 0.5 M H_2_SO_4_ electrolytes, HER overpotentials were 31 mV and 148 mV. The Tafel slopes of HER and OER at 1.0 M KOH were 30 mV·dec^−1^ and 51 mV·dec^−1^, respectively. Thus, FeCoNiB@B-VG has a large range of PH applicability.

In addition, Ye et al. [120] used plasma etching to generate additional topological defects on S-doped graphene and the synergistic interaction between S-doping and plasma-induced surface defects resulted in plasma-etched S-doped graphene showing significantly enhanced HER activity at current densities, with a low overpotential of 178 mV and a Tafel slope as low as 86 mV dec^−1^ at a current density of 10 mA·cm^−2^. Recently, Sun et al. [121] used the one-pot method to fabricate ultra-small ruthenium nanoparticles (NP) dispersed on S-doped graphene (Ru/S-RGO). The presence of S enhanced the uniform dispersion of ruthenium nanoparticles and exposed more active sites. At a current intensity of 20 mA·cm^−2^, the prepared Ru/S-RGO-24 exhibits an overpotential as low as 14 mV showing good HER catalytic performance.

#### 4.2.2. 3D-Type Catalysts

Also for 3D-type electrocatalysts prepared from single-element doped graphene, Zang et al. [122] prepared an N-doped graphene derivative of MoS_2_ (MNG) with an integrated metal-semiconductor (1T-2H) hybrid phase having a three-dimensional porous structure using urea as a dopant. Under acidic conditions, MNG-40 exhibited a low overpotential of 157 mV and a Tafel slope of 45.8 mV·dec^−1^, with good HER catalytic activity.

Ion-Ebrașu et al. [123] prepared three-dimensional graphene foam (3D-GrFoam) with high pore structure by CVD and used a hydrothermal method to functionalize the 3D-GrFoam with nitrogen to synthesize a non-precious metal HER catalyst with good catalytic activity in alkaline media.

Shen et al. [124] investigated the effect of different nitrogen precursors on the structure of nitrogen-doped 3D spherical hollow graphene and the changes in HER based on this nitrogen-doped graphene structure. The results showed that the N-doping site can act as a nucleation and growth center for MoS_2+x_. Different nitrogen precursor dopants have a significant influence on the shape and structure of the catalyst loaded with MoS_2+x_.

In addition to nitrogen-atom-doped graphene, P-atom doping is a promising strategy for promoting HER activity. Yu et al. [125] proposed a new method for fabricating one-dimensional hollow transition metal phosphides using the electrostatic repulsion-limited nucleation (ERLN) policy in which arrays of small-sized FeOOH nanorods grown on P-heteroatom-doped graphene were further phosphorylated in situ into FeP nanotubes, to produce P-doped G-supported FeP nanotube arrays composed of 3D porous structures. The electrocatalyst exhibited outstanding HER catalytic performance with overpotential of 69 mV and a Tafel slope of 52.4 mV·dec^−1^ at a current density of 10 mA·cm^−2^.

Li et al. [126] successfully laminated P-Fe_3_O_4_ on 3D porous graphene foam by hydrothermal and phosphorylation reactions to produce P-Fe_3_O_4_ @3DG composites, which exhibited outstanding HER catalytic activity and long-term endurance in acidic conditions. The P doping resulted in a lower binding energy of H^+^, which promoted H^+^ adsorption and kinetically facilitated the catalytic process.

#### 4.2.3. Dual-Doped Graphene Powder Catalysts

N and P have the same number of valence electrons as well as similar chemical properties [127]. Based on the synergistic effect that activates more adjacent C atoms to enhance their catalytic activity, the two-element doped graphene-based electrocatalysts exhibit significant catalytic activity for the decomposition of water. Based on this, Yaroslav et al. [128] obtained a catalytic PAIn-PMo12/rGO electrocatalyst with good catalytic performance based on the active sites in the carbon-based composite and the possible synergistic interactions between them by high-temperature processing of a mixture of PAIn, PMo12, and rGO precursors. The catalyst was effective in promoting HER production over a broad pH spectrum, and the Tafel slopes on Mo2C, Mo2N/N, and P-rGO reached 60 and 57 mV·dec^−1^ at 10 mA·cm^−2^ in 0.5 M H_2_SO_4_ and 1.0 M KOH, corresponding to overpotentials of 195 and 115 mV, respectively.

The reaction kinetics of HER in alkaline dielectrics is slower and requires more energy to facilitate the dissociation of water molecules. To this end, Chen et al. [129] supported MoP nanoparticles optimized by W atoms on N,P-doped multilayer graphene oxide to obtain HER electrocatalysts, W_0.25_Mo_0.75_P/PNC with high catalytic activity, and very low overpotential. DFT calculations showed that the W_0.25_Mo_0.75_P/PNC interfacial site could significantly reduce the potential barrier during water electrolysis, improving the HER kinetic conditions (η = 70 mV@10 mA·cm^−2^, η = 49 mV@10 mA·cm^−2^).

Sun et al. [130] anchored doubly zero-valent N,B-doped Ni_2_P nanoparticles onto N,B co-doped graphene by in situ assembly to obtain electrocatalysts with substantially increased HER catalytic activity at all pH conditions, and further demonstrated that the synergistic interaction between the zero-valent N, B heteroatom co-doping optimized the free energy of water and hydrogen adsorption by density functional theory (DFT).

**Figure 6:**
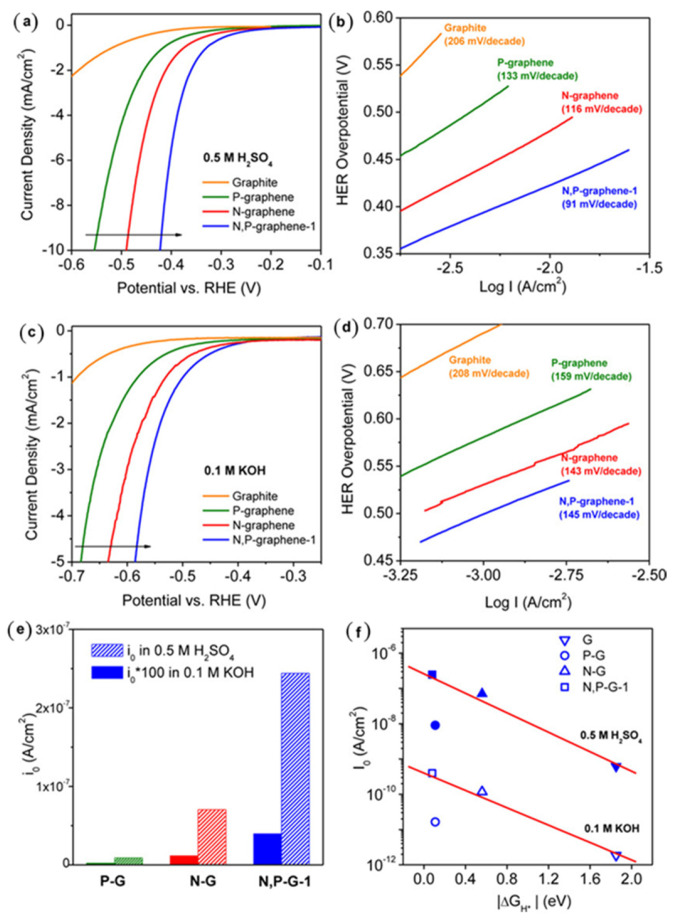
HER polarization curves and respective Tafel plots of N− and/or P−doped graphene electrocatalysts in (**a**,**b**) 0.5 M H_2_SO_4_ (pH = 0) and (**c**,**d**) 0.1 M KOH (pH = 13). (**e**) The calculated values of i0 for N and/or P−doped graphene in 0.5 M H_2_SO_4_ (patterned bars) and 0.1 M KOH (solid bars) solutions are compared (*100 is 100 times than original data). (**f**) Relationship between exchange current density (I_0_) and free energy (ΔG_H*_) of N− and/or P−doped graphene. Reprinted with permission from Ref. [131]. Copyright 2014 American Chemical Society.

Zheng et al. [131] designed and synthesized nitrogen (N) and phosphorus (P) double-doped graphene as a non-metallic electrocatalyst for sustainable and efficient hydrogen production. The analytical results showed that the double-doped graphene showed higher electrocatalytic HER activity than the single-doped graphene and the performance was comparable to some conventional metal catalysts (Figure 6).

In addition, Subhasis et al. [132] also investigated the effect of simultaneous or separate doping of N and B on the catalytic performance of rGO. The doping of N and B in reduced graphene oxide (rGO) changed the lattice parameters and electronic structure of rGO, promoting the adsorption and desorption of the substrate and modulating the catalytic activity of rGO towards HER.

## 5. Graphene Quantum Dot Catalysts

Graphene quantum dots (GQD) are important derivatives of graphene. GQDs are zero-dimensional graphene sheets with unique properties influenced by oxygen-rich functional groups at the edges compared to other carbon nanomaterials. GQDs have tunable physical properties and high chemical stability, and GQDs with diameters less than 2 nm allow for higher specific surface areas, abundant edge defects, and high surface activity. In addition, GQDs can be used to enhance electrocatalysis by increasing the interspacing. GQDs are of increasing interest to researchers as they can enhance electrocatalysis by increasing interspacing and defects and enabling many electron and ion transfer mechanisms [132,133,134].

For example, Guo et al. [135] prepared MoS_2_@graphene functionalized N-doped graphene quantum dots (MoS_2_@N-GQDs-GR) using a single-pot hydrothermal approach. HER catalytic activity with excellent long-term stability even after 3000 consecutive scans was observed. It is reported that the doping of GQD effectively enhances the adsorption of H by S atoms in MoS_2_ nanosheets, and the very large two-dimensional conducting properties and large surface area of N-GQDs-GR allow the HER electrocatalyst to exhibit significant catalytic efficiency with low overpotential and low Tafel slope.

Yelyn et al. [136] published the synthesis of nitrogen and fluorine co-doped graphene quantum dots (N,F-GQDs) by a dual-step functionalization approach. This involved the reaction of ammonia (NH_3_) gas with GQDs by chemical vapor deposition to prepare N-GQDs, followed by fluorination operations to finally obtain N,F-GQDs composites, which exhibited significant catalytic activity at an overpotential of 10 mA·cm^−2^ for HER. The composite exhibited significant catalytic efficiency with an overpotential of 0.13 V at a current intensity of 10 mA·cm^−2^ during aqueous decomposition. Graphene oxide quantum dot-MoS_x_ nanohybrids (GOQD-MoS_x_) prepared by a novel method were reported by Marco et al. [137]. The findings suggest that the growth of GOQDs inhibited the growth of MoS_2_NPs, resulting in a large number of exposed edge sites dispersed in the GOQDs matrix. The edges of GOQD-MoS_3_ nanohybrids encountered a sulfur S coordination change from S-dimers (S_2_^2−^) to S-monomers (S^2−^), with the change in S coordination having a significant effect in the activity of these materials. Xiao et al. [138] prepared Pt and CQDs co-loaded graphene catalysts by an electrolytic-solvothermal method. The large active surface area and abundant defects possessed by the catalysts resulted in enhanced HER catalytic activity in acidic dielectrics. Overall, excellent HER catalytic activity at −50 mV with a mass activity of 37.5 A mg^−1^, which is 68.2-fold more active than Pt/C, was observed.

Similarly, since the unique properties of 2D materials for energy conversion, catalysis, optoelectronics, and thermoelectric devices could change the electrocatalytic landscape, but traditional chemical vapor deposition methods are costly and unsuitable for mass production, Gong et al. [139] proposed a new strategy using pyrene-grown graphene quantum dots as dispersant and embedding agent in a water/ethanol mixture. The graphene quantum dots were exfoliated from atomically thin 2D materials (MoS_2_, h-BN, WS_2_, gC_3_N_4_ microsheets) by sonication for 8 h prior to centrifugation. The GQD/MoS_2_ van der Waals heterojunctions (vdWHs) formed in this study exhibited significant enhancement in the electrocatalytic performance (HER) due to the synergistic coupling at the 0D/2D heterojunctions.

### 5.1. 3D-Type Catalysts

Li et al. [140] exploited the susceptibility of yeast cells to adsorb nickel ions (Ni^2+^) by embedding Ni-Ni_3_P nanoparticles on a three-dimensional graphene backbone co-doped with N and P. The resulting Ni-Ni_3_P@NPC/rGO electrocatalyst had a low grade overpotential of 113 mV (up to 20 mA·cm^−2^) and Tafel slope of 57.93 mV·dec^−1^, with good HER catalytic properties. In addition, the analytical results show that the Ni/Ni_3_P heterostructure can also attenuate the strong Ni-H bonding to obtain a small Δ_GH_*.

Zhao et al. [141] successfully synthesized heteroatom-doped 3D carbon nanotube/graphene hierarchies (N,S-CNTs/N,SG) by two different CVD methods using layered dioxides as substrates. The analysis showed that the catalysts contained high levels of N and S dopants, which provided more active sites, and had a low starting potential in 0.5 M H_2_SO_4_ (62 mV vs. RHE) along with a low overpotential of 126 mV at a current intensity of 10 mA/cm^2^, with significant HER catalytic efficiency. Wang et al. [142] reported two Co and N co-doped 3D graphene materials, which were prepared by a single-step simultaneous doping approach (Co,N/3DG-1) and a double-step order doping strategy (Co,N/3DG-2), respectively. The analysis results showed that the catalysts obtained by sequential doping had better catalytic activity. In addition, DFT calculation and experimental results show that sequential doping may make the obtained Co-N more moderate and coordinated.

N, S co-doped graphene-based catalysts are a powerful alternative to precious metal electrocatalysts for HER reactions. Ceren et al. [143] have recently successfully decorated CoP nanoparticles into 3D graphene co-doped with nitrogen and sulfur by hydrothermal auto-assembly and the cryogenic phosphorylation route. The prepared CoP@N,S-3D-GN composites have a core/shell morphology, which allows the catalyst to have prolonged stability and good HER catalytic performance, exhibiting an overpotential as low as 118 mV and a low Tafel slope (50 mV·dec^−1^) at a current intensity of 10 mA/cm^2^.

### 5.2. Dual-Action Catalysts

Graphene-based dual-functional electrocatalysts for overall water splitting (OWS) are a promising technology for the mass production of H_2_ and O_2_, including oxygen evolution reactions with four electronic transfers at the anode, and hydrogen evolution reactions with two electron transfers at the cathode. Since the four-electron transfer in the OER process is slow, the efficiency of overall water separation is restricted by the efficiency of OER electrocatalyst.

Recently, to improve the electrocatalytic efficiency of NiFe_2_O_4_, D. Navadeepthy et al. [144] used a hydrothermal method to decorate N-doped graphene on the original catalyst to produce nanocomposite N-doped graphene/NiFe_2_O_4_ electrocatalyst (NGNF). At a current intensity of 10 mA/cm^2^, the HER and OER exhibited lower overpotentials of 184 mV and 340 mV and Tafel slopes of 82.9 and 93.2 mV·dec^−1^. The results suggest that the improved catalytic properties are attributed to the increased surface area of the N-doped graphene for the catalysts and the increased catalytic activity.

Previously, Hu et al. [145] reported a bifunctional electrocatalyst for monolithic hydrolysis, NG-NiFe@MoC_2_, which exhibits small charge transfer resistance and high stability as well as electrocatalytic synergy, resulting in excellent catalytic performance in HER or OER as well as OWS. It is remarkable that the catalyst needs a low voltage of 1.53 V to reach a 10 mA/cm^2^ current intensity which is considerably preferable to most nonprecious-metal-based catalysts and the expensive Pt/C//RuO_2_ electrocatalysts (1.61 V).

Bu et al. [146] reported a new route for microwave-assisted heat conversion to achieve ultra-rapid synthesis of FeNiP/graphene composites after 20 s of 1000 W microwave irradiation and revealed that transition metal phosphines and their derived oxides/hydroxides are the most reactive sites for HER and OER, respectively, for the first time. This bimetallic phosphide/graphene heterostructure provides very low overpotentials of ~229 and 173 mV for OER and HER at a current intensity of 10 mA/cm^2^, exhibiting remarkable catalytic properties for the electrochemical decomposition of water.

**Figure 7:**
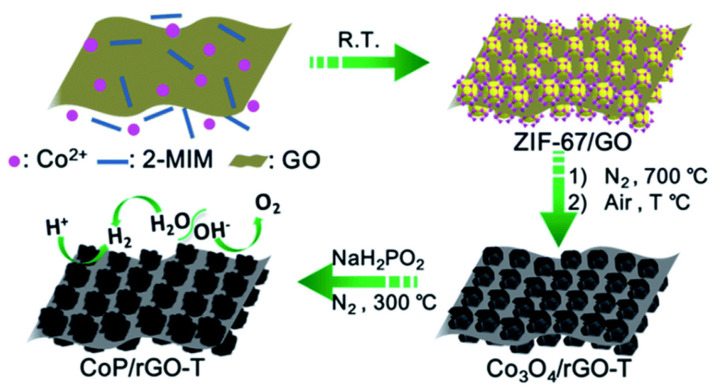
Description of the fabrication process of CoP/rGO-T nanocomposites. Reprinted with permission from Ref. [147]. Copyright 2016 The Royal Society of Chemistry.

Jiao et al. [147] successfully prepared layered CoP/reduced graphene oxide (rGO) composites using a rationally designed sandwich-type metal-organic backbone/graphene oxide as a template and precursor (Figure 7). Since the material was in alkaline solution, it exhibited excellent catalytic properties for both HER and OER. Therefore, it can be used for monolithic hydrolysis in alkaline media and even exhibits better catalytic activity than Pt/C and IrO.

Sun et al. [148] reported a new CoP@N,P C composite with a 3D porous structure prepared by a one-step pyrolysis method, which has excellent overall hydrolytic catalytic performance in the electrochemical decomposition of water. The analysis results indicated that it is this 3D porous structure that offers more active points for the catalytic procedure and promotes ion transport efficiency during the reaction. In addition, there is a synergistic effect between the co-doped carbon of N and P and the CoP nanoparticles, improving the electrical conductivity and catalytic activity of the matrix. The long-term stability of the system was maintained after 5000 cycles of electrochemical experiments using this catalyst CoP@N,P C.

Moreover, in the exploration of 3D-type electrocatalysts, Nabi et al. [149] reported a novel 3D graphene-decorated CoO nanoparticle electrocatalyst in which graphene flakes act as carriers for CoO particle dispersion and rapid electron transport, while the CoO particles act as active sites for the OER and HER, which are significantly reactive to enhance the overall hydrolysis process.

**Table 1:** Summary of HER performance of graphene-based electrocatalysts.

Electrocatalyst	Electrolyte	Onset Potential/Overpotential	Tafel Slope (mV·dec^−1^)	Stability	Ref.
ReSe_2_ nanoflakes/rGO	0.5 M H_2_SO_4_	145.3 (vs. REH) at 10 mA cm^−2^	40.7	24 h	Yan et al. [86]
Ni_0.85_Se nanospheres/rGO	1 M KOH	128 (vs. REH) at 10 mA cm^−2^	91	18 h, 1000 CV	Zhu et al. [91]
Conducting scaffold-supported 3D rGO-CNT/MoS_2_ nanostructure	0.5 M H_2_SO_4_	123.75 (vs. REH) at 100 mA cm^−2^	31		Bolar et al. [96]
	1 M KOH	217.61 (vs. REH) at 50 mA cm^−2^	52		
3D Pd nanosponge-shaped networks wrapped by graphene dots	0.5 M H_2_SO_4_	32 (vs. REH) at 10 mA cm^−2^	33	15 h, 3000 CV	Nguyen et al. [99]
3D graphene hollow nanospheres supported ruthenium phosphides	1.0 M KOH	25.5 (vs. REH) at 10 mA cm^−2^	34.4	10 h, 1000 CV	Li et al. [101]
Ru Nanoclusters/N-graphene	1.0 M KOH	25.9 (vs. REH) at 10 mA cm^−2^	32.6	2000 CV	Li et al. [113]
FeCoNiB@Boron-doped vertically aligned graphene arrays	1.0 M KOH	31 (vs. REH) at 10 mA cm^−2^	30	10 h	Jiang et al. [119]
Plasma-etched, S-doped graphene	0.5 M H_2_SO_4_	178 (vs. REH) at 10 mA cm^−2^	86	20 h, 3000 CV	Tian et al. [120]
3D porous NG derivative-integrated MoS_2_ nanosheet	0.5 M H_2_SO_4_	157 (vs. REH) at 10 mA cm^−2^	45.8	10 h, 1000 CV	Zang et al. [122]
3D FeP NT/PG	0.5 M H_2_SO_4_	69 (vs. REH) at 10 mA cm^−2^	52.4	40 h, 1000 CV	Yu et al. [125]
A self-supporting P–Fe_3_O_4_@3DG bulk composite	1.0 M KOH	123 (vs. REH) at 10 mA cm^−2^	65	50 h	Li et al. [126]
Ni_2_P nanoparticles/N,B-graphene	1.0 M KOH	92 mV (vs. RHE) at 10 mA cm^−2^	48.3	20 h, 3000 CV	Sun et al. [130]
Dispersed tungsten (W)-optimized MoP nanoparticles on N,P-doped graphene oxide	1.0 M KOH	70 mV (vs. RHE) at 10 mA cm^−2^	60.3	16 h	Chen et al. [129]
Ni-Ni_3_P@NPC/rGO	0.5 M H_2_SO_4_	113 mV (vs. RHE) at 20 mA cm^−2^	57.93 mV dec^−1^	25 h	Li et al. [140]
Cobalt phosphide decorated/N,B-3D-graphene	0.5 M H_2_SO_4_	118 mV (vs. RHE) at 10 mA cm^−2^	50	50 h, 1000 CV	Karaman et al. [143]

## 6. Conclusions and Future Perspectives

With high prices and scarce resources for rare precious metal catalysts, there is an immediate need to find a highly efficient, low-cost HER electrocatalyst to meet current demand.

Advanced graphene-based materials have been widely used in the electrocatalytic decomposition of water because of their high electronic conductivity, diverse morphology, abundant and economical sources of raw materials, and environmental friendliness. This paper systematically reviews the recent research progress on graphene-based materials as HER electrocatalysts. In the previous discussion, various effective preparation methods and design strategies have been discussed, including strategies employing heteroatom doping, graphene-encapsulated metal nanoparticles, defect engineering, surface functionalization, edge clipping, construction of 3D morphologies, and fabrication of porous structures; in addition, some promising HER electrocatalysts with comparable commercial catalysts have been reported. Among them, the most widely used are various doped graphene-based electrocatalysts.

Although major advances have been made with graphene-based HER catalysts, many challenges remain to be solved. Firstly, although recently developed novel HER electrocatalysts have shown considerable activity towards HER, the catalytic performance of these catalysts is still below the performance of typical Pt/C electrocatalysts, so there is still much scope to increase the catalytic performance further. Secondly, the lack of precise control over the composition, architecture, and activity sites of many graphene-based materials may result in the loss of their unique structural benefits to the extent that a significant proportion of the electrochemical active sites are covered, thereby reducing the performance of the final electrocatalyst. Thirdly, as more and more heteroatoms are doped with more and more elements, there is less and less incentive provided in terms of heteroatom doping alone to enhance catalytic activity. However, the scope for research contained therein is enormous for defect engineering and this is an area that should perhaps be explored intensively in the future. Fourthly, DFT calculations have influenced the design of the activity of new electrocatalysts. Without the help of theoretical calculations, it is difficult to understand and grasp the mechanism of operation of HER electrocatalysts and the relationship between activity and stability during the electrolysis process. However, the mechanisms and active sites in some reactions are still not conclusive and a deeper understanding of these theories will be required to design new HER electrocatalysts in the future.

In summary, graphene-based catalysts have a promising future as emerging electrocatalysts for water electrolysis, with both opportunities and challenges. Even this short review reveals the importance of graphene in HER electrocatalysis in the future.

## Data Availability

Not applicable.

## Abbreviations

OER, oxygen evolution reaction; HER, hydrogen evolution reaction; CNTs, carbon nanotubes; CQDs, carbon quantum dots; MWCNT, multiwall carbon nanotube; HTC, hydrothermal carbonization; NSG, N,S co-doped graphene; GQDs, graphene quantum dots; TMC, transition metal carbides; CVD, chemical vapor deposition; GO, graphene oxide; MOFs, metal-organic frameworks; ΔG, free energy; DFT, density functional theory.
